# Oscillatory Correlates of Intentional Forgetting: The Role of Theta and Alpha Power in Item-Method Directed Forgetting

**DOI:** 10.1523/ENEURO.0022-21.2021

**Published:** 2021-10-08

**Authors:** Sebastian Scholz, Stephan Dutke, Niko A. Busch

**Affiliations:** 1 Institute for Psychology in Education, University of Münster, 48149 Münster, Germany; 2 Institute for Psychology, University of Münster, 48149 Münster, Germany

**Keywords:** alpha power, directed forgetting, EEG oscillations, recognition memory, theta power

## Abstract

Results from item-method directed forgetting suggest that individuals are able to intentionally forget processed information. Most research suggests that either selective rehearsal of to-be-remembered or inhibitory control of to-be-forgotten information is accountable for the effects of intentional forgetting. Some research, however, hypothesized that the time to process information mediates the underlying mechanism. To test this hypothesis, the current study investigated associations between oscillatory power in theta (3–7.5 Hz) and alpha frequencies (8–13 Hz) and intentional forgetting in human participants and explored whether or not these mechanisms depended on processing time. Previously, theta power was shown to be associated with the creation of episodic memory traces and alpha power with inhibition. We therefore expected to find associations between these neural signatures and behavioral effects. Consistent with our hypotheses, we revealed increased theta power for to-be-remembered and increased alpha power for to-be-forgotten information and that the effects of activity in both frequency bands were influenced by the time individuals were given for processing the memory cue. These results suggest that not one but two mechanisms, rehearsal and inhibitory control, are accountable for item-method directed forgetting, both with different temporal profiles.

## Significance Statement

We investigated oscillatory activity in the EEG while participants completed an item-method directed forgetting task, in which the time given to process remember/forget instructions was varied. Our results revealed increased theta (3–7.5 Hz) and alpha (8–13 Hz) power for successfully forgotten information. Contrary to most research (Anderson and Hanslmayr, 2014), the reported pattern of results suggests a two-process account of directed forgetting. This account involves a faster rehearsal stage, which is associated with increased theta power, and a slower inhibitory control stage, which is associated with increased alpha power. Furthermore, we presented evidence that these processes were influenced by the time given to process remember/forget instructions, which is in line with behavioral studies (Wetzel and Hunt, 1977; Fawcett and Taylor, 2008).

## Introduction

Forming strong memories requires not only efficient encoding of relevant information, but also efficient forgetting of irrelevant information to avoid interference between memories (Bjork, 1970). Forgetting has been studied, for example, with the item-method directed forgetting (DF) paradigm (Basden et al., 1993; MacLeod, 1998), in which participants receive one stimulus at a time followed by a cue instructing them to either remember or forget that stimulus. After several of these stimulus–cue combinations, a final memory test typically reveals that more stimuli cued to remember than cued to forget are recalled (Fawcett and Taylor, 2008). Two theoretical accounts have been put forward to explain this result (Anderson and Hanslmayr, 2014). According to the selective rehearsal account (Greene, 1987), to-be-remembered (TBR) stimuli are actively rehearsed in working memory, while to-be-forgotten (TBF) stimuli are excluded from rehearsal and are subject to passive forgetting. This explanation was supported by prior electroencephalographically (EEG) research (Hsieh et al., 2009). By contrast, the inhibitory control account assumes that TBF stimuli are actively inhibited and deprived of attention (Zacks et al., 1996; Fawcett and Taylor, 2008). In line with this account, several studies have demonstrated that directed forgetting might be more cognitively demanding than remembering. It involves active attentional withdrawal as indicated by stronger inhibition-of-return (Fawcett and Taylor, 2010) and eye movements directed away from TBF stimuli (Lee, 2018). In addition, functional magnetic resonance imaging (fMRI; Wylie et al., 2008) and EEG studies (Ullsperger et al., 2000; van Hooff and Ford, 2011) have demonstrated that directed forgetting engages active inhibition during encoding. Oscillatory power in the alpha band (8–13 Hz) is of particular interest here, since a large body of literature has demonstrated that alpha power is related to neural inhibition and suppression of cognitive processing (Kelly et al., 2006; Klimesch et al., 2007; Jensen and Mazaheri, 2010; Foxe and Snyder, 2011; Park et al., 2014; Samaha et al., 2020). In other memory tasks, strong alpha power during encoding predicts forgetting at test (Hanslmayr et al., 2012; Fellner et al., 2013). Oscillatory activity in the theta band (3–7.5 Hz) might also be related to memory processes. Increased theta activity was reported when encoding resulted in successful retrieval, memory maintenance, and rehearsal (Klimesch et al., 1996; Sederberg et al., 2003; Lega et al., 2012). Activity in these frequency bands might therefore indicate different cognitive processes in DF.

Recent work suggested that both selective rehearsal and inhibitory control may contribute to forgetting in DF. Fellner et al. (2020) revealed that successfully forgotten TBF stimuli were associated with an early (0.5 s after cue) increase in alpha power, indicating stronger inhibition, and with a late (1.5 s after cue) decrease in alpha power, indicating reduced rehearsal. Thus, both mechanisms may be related to directed forgetting, albeit with different temporal profiles.

Time-critical aspects might influence DF even more directly. Behavioral studies suggested that the processing of TBR and TBF information might depend on the available processing time following the memory cue and that only TBR stimuli benefited from additional processing time (Wetzel and Hunt, 1977). Fawcett and Taylor (2008) reported that only short (1.4 s) but not longer (2.6 s) processing times resulted in increased forgetting demands, which are indicated by longer reaction times in a secondary task. To investigate the relationship between processing time and oscillatory activity directly, we measured EEG while participants performed an item-method DF task in which the time to process the cue was varied blockwise (1 vs 3 s).

Next to a general behavioral directed forgetting effect, we expected that long intervals improve memory performance specifically for TBR stimuli. We also expected stronger theta power following TBR cues, indicating selective rehearsal, and stronger alpha power following TBF cues, indicating the engagement of inhibition. Furthermore, we expected stronger theta power and less alpha power following subsequently remembered stimuli than subsequently forgotten stimuli. Specifically, we expected the strongest theta power for subsequently remembered TBR stimuli, and the strongest alpha power for subsequently forgotten TBF stimuli. Finally, we expected that the differences in the theta and alpha band depend on the time given to process the memory cue, indicating that the available processing time mediates the degree of rehearsal and inhibition in the control of memory encoding.

## Materials and Methods

### Participants

In total, 45 human participants performed the task. However, the data of three participants were excluded from the dataset (2 participants had a false alarm rate as high as the hit rate and 1 participant misunderstood the task). The remaining 42 participants (32 females) were within an age range of 18–29 years (mean* *=* *23.5 years, SD* *=* *3.1) and completed the experiment for either course credits or a monetary reward. All participants were university students, native speakers of German, had normal or corrected visual acuity, no history of past neurologic or psychological disorders, and were right handed. All procedures were performed in accordance with the Declaration of Helsinki and were reviewed by the university ethics committee.

### Design and stimuli

The experiment consisted of an encoding phase, a distraction task, and an old–new recognition test. The experiment was expressed as a 3 (cue: TBR vs TBF vs Neutral) × 2 [interstimulus interval (ISI) duration: 1 vs 3 s] within-participants design with recognition accuracy as the dependent variable. Accuracy was assessed using a confidence measure (six levels, ranging from “very certain that the stimulus is new” to “very certain that the stimulus is old”). ISI durations varied blockwise with two blocks for each duration. The order of blocks was randomly permuted between participants. For later EEG analyses, memory outcome was categorized as subsequently remembered or subsequently forgotten. A stimulus was regarded as remembered if participants pressed one of three keys associated with “old” when an old stimulus was presented during encoding. In contrast, a stimulus was categorized as subsequently forgotten if participants pressed one of the three keys associated with “new” although an old stimulus was presented.

Stimuli were taken from the Berlin Affective Word List (Võ et al., 2006) that includes 2902 German words characterized by different variables taken from the CELEX database (Baayen et al., 1995). Parameters were set to include only nouns that were four to eight characters long with an average word frequency ± 1.5 SDs, emotional valence between +1 and −1, and average imaginability and arousal ratings ± 1.5 SDs. The application of these parameters resulted in a word pool of 543 words from which 480 words were randomly drawn for each participant and split into two sets. One set was presented during encoding, words from the other set served as distractors during old–new recognition. The word set for the encoding phase (240 words in total) was randomly split into four subsets, two sets for the short ISI (1 s) and two for the long ISI (3 s; 120 words each). Each of the four sets was randomly split into three subsets for the three instructions: TBR, TBF, and Neutral with 40 words each. All lists were randomly permuted between participants.

Stimuli were presented in front of a gray background in the center of the screen in black font color on a PC running Windows 10 using MATLAB (version 2018a) and Psychtoolbox (version 3.0.15). While completing the experiment, participants sat 60 cm away from a 17 inch computer screen.

### Procedure

#### Encoding phase

Participants filled out a demographic questionnaire, gave written informed consent, and read the instructions for the experimental task, which emphasized that only the words that were followed by a TBR instruction should be recalled in the recognition test at the end of the experiment. Furthermore, it was emphasized that a huge number of trials will be presented and that not all information can be remembered. This instruction was included to simultaneously increase rehearsal processes following TBR cues and to prevent the learning of TBF stimuli. Then, the EEG system was applied, and participants completed three practice trials, one for each condition, after the remaining questions were answered. The encoding phase started and ended with two additional trials that were instructed to be forgotten and not part of the analyses. These four trials were included to prevent potential primacy and recency effects.

**Figure 1.**
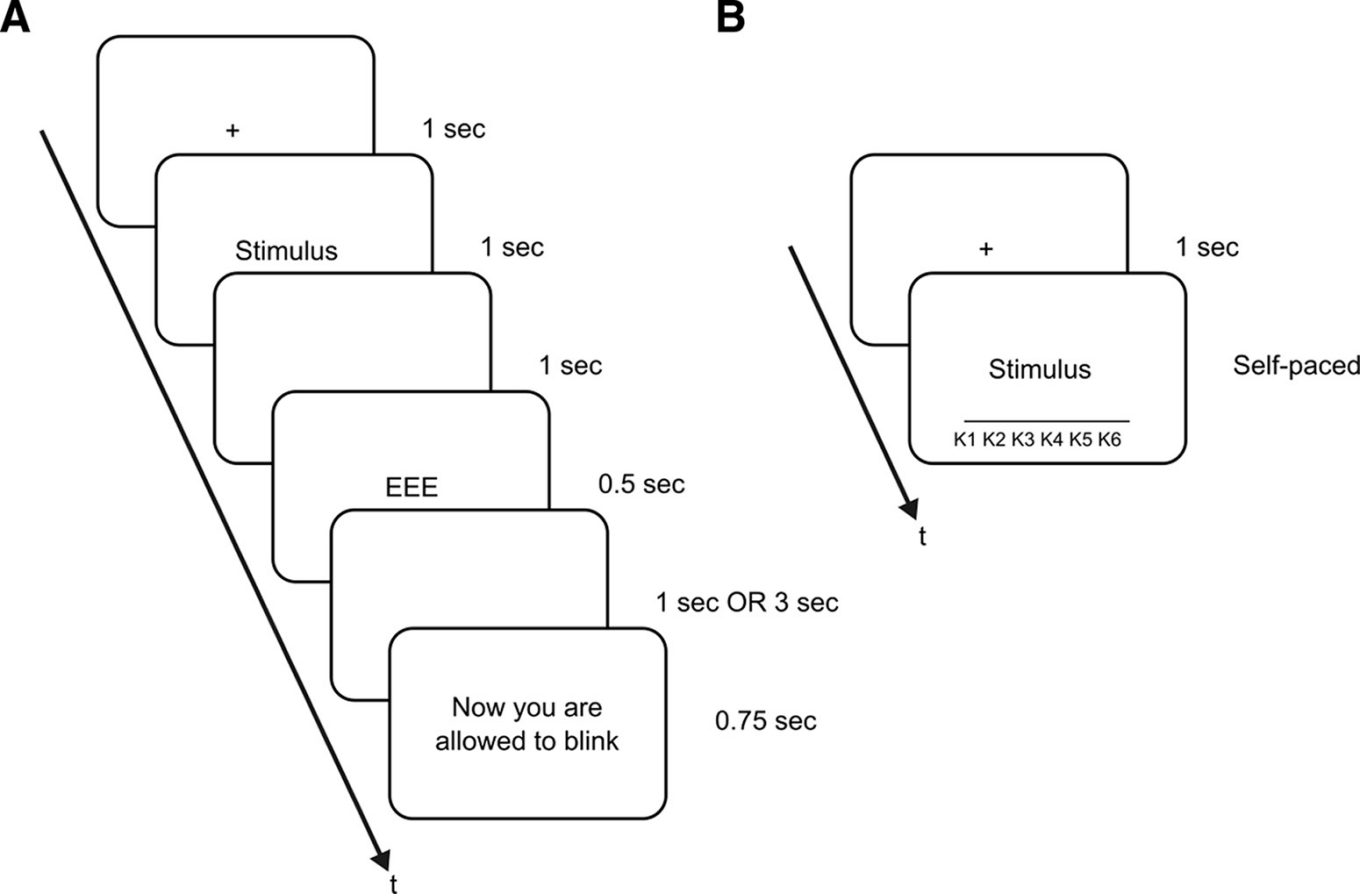
Depiction of the experimental task. ***A***, In the encoding phase, the duration of the second ISI depended on the experimental block. Cues were EEE (TBR), VVV (TBF), or NNN (Neutral). ***B***, Old–new recognition test during which participants pressed one of six keys dependent on their confidence ranging from “very certain that an item is new” (K1) to “very certain that an item is old” (K6).

Each trial of the encoding phase started with a fixation cross for 1 s, followed by the word for 1 s, an ISI for 1 s, the cue (EEE = TBR; VVV = TBF; NNN = Neutral) for 0.5 s, and an ISI for either 1 s (short ISI) or 3 s (long ISI) depending on the experimental block. We included the Neutral condition to test the participant’s behavior when no precise memory instruction was given (Gao et al., 2016; Scholz and Dutke, 2019). Each trial ended with a 0.75-s-long intertrial interval in which participants were allowed to blink. Therefore, the total duration of each trial was either 5.25 s (short ISI) or 7.25 s (long ISI; Fig. 1*A*). Following each block of 60 trials, participants had a 45-s-long resting phase before they were informed about the duration of the ISI in the next experimental block. To continue with the next block, participants pressed the space bar. On average, the encoding phase lasted for 29 min.

#### Distraction task

To prevent internal rehearsal following the encoding phase, a distraction task was included in which participants had to spot and mark differences in two puzzle pictures for 5 min.

#### Old–new recognition test

For memory testing, participants completed an old–new recognition test, in which the words from the encoding phase and new distractor words were presented in random order. Each trial started with the presentation of a fixation cross for 1 s, followed by the stimulus (Fig. 1*B*). Participants were instructed to press one of six buttons, depending on whether they judged the stimulus to be old or new and how much confidence they had in the correctness of their answer. Each key press was immediately followed by the next trial. Participants were advised to work as fast and precisely as possible. Following the presentation of 80 trials, short breaks were included that were manually terminated by pressing the space bar. In total, 480 trials were presented, and the recognition test lasted on average for 23 min.

### EEG data collection

EEG activity was measured with 32 active electrodes at locations based on the 10–20 system (ActiCap, Brain Products). All electrodes were online referenced to FCz, and all impedances were kept to <20 kΩ. EEG data were collected using BrainVision Recorder (version 1.21.0102) running on a Windows 10 PC. During data digitization and amplification, two online filters, one with a low cutoff frequency of 0.531 Hz and one with a high cutoff frequency of 100 Hz, were applied. Data were sampled with a sampling frequency of 500 Hz, and all of the following EEG analyses were performed with MATLAB (R2018b) using self-programmed routines and procedures implemented in the FieldTrip toolbox (http://www.ru.nl/fcdonders/fieldtrip).

### EEG data preprocessing and time–frequency decomposition

The continuous EEG data were separated in 240 trials epoched around cue onset (−2.5 to 1.5 s) and rereferenced to the average of all EEG channels. Independent component analyses (ICAs) were conducted to correct for residual artifacts in the trials (e.g., eye blinks or eye movements). Visually identified components were discarded from the data (mean* *=* *1.5 components, SD* *=* *0.8). Then, trials were separated for the three cues (TBR, TBF, and Neutral), the subsequent memory outcome (remembered or forgotten), and the duration of the ISI (short or long). To be able to compute interaction effects, data were also separated for combinations of ISI and cue. We used an automatic artifact correction to exclude trials with artifacts other than those detectable using an ICA. Complete trials were automatically rejected based on amplitude (±150 μV). On average, the following number of trials passed the artifact rejection: TBR: mean* *=* *78.5; range = 58–80; TBF: mean* *= 78.5; range = 55–80; Neutral: mean* *=* *78.4; range = 53–80; subsequently remembered: mean* *=* *160.2; range = 105–214; subsequently forgotten: mean* *=* *75.2; range = 26–135; short ISI: mean* *=* *118.1; range = 99–120; long ISI: mean* *= 117.3; range = 67–120; subsequently remembered TBR: mean* *=* *62.1; range = 37–78; subsequently remembered TBF: mean* *=* *48.5; range = 29–64; subsequently remembered Neutral: mean* *=* *49.6; range = 26–73; subsequently forgotten TBR: mean* *=* *16.3; range = 2–37; subsequently forgotten TBF: mean* *=* *30.0; range = 15–51; subsequently forgotten Neutral: mean* *=* *28.8; range = 7–51; short ISI TBR: mean* *=* *39.3; range = 35–40; short ISI TBF: mean* *=* *39.4; range = 32–40; short ISI Neutral: mean* *=* *39.5; range = 32–40; long ISI TBR: mean* *=* *39.2; range = 23–40; long ISI TBF: mean* *=* *39.1; range = 23–40; and long ISI Neutral: mean* *=* *39.0; range = 21–40. Participants with <10 trials in one condition (nine participants for forgotten TBR trials) were excluded from the analyses using this condition (difference between remembered TBR and remembered TBF stimuli, and difference between forgotten TBF and forgotten TBR stimuli). For further processing steps, data from all trials in one condition were averaged for each participant to adjust for differences in the number of trials.

Time–frequency decomposition was accomplished using a wavelet convolution with a fixed Hanning window length of 500 ms. Frequencies of interest were chosen between 1 and 30 Hz with bins of 0.5 Hz and time points of interest for the complete epoch (−2.5 to 1.5 s around cue onset) with time bins of 100 ms. We extracted power values, and time–frequency power data were baseline corrected using the relative change from processing the fixation cross (−2.5 to −2 s before cue onset) to the complete epoch.

### Statistical analyses

#### Behavioral analysis

Differences in recognition performance were investigated using signal detection theory (SDT; Green and Swets, 1966). The SDT framework presumes that the strength of a continuous signal (e.g., memory strength) varies on a continuum, and a participant has to decide when a threshold is exceeded to decide between two options (e.g., old and new in recognition experiments). As a dependent variable, we chose the area under the receiver operator characteristic (ROC). The area under the ROC represents a nonparametric characteristic for discriminability and indicates the relationship between the cumulative increase of hit rates (answering “old” for presented stimuli) and the cumulative increase of false alarm rates (answering “old” for new words) with decreasing confidence in the given answer. The larger the area under the ROC, the better the discrimination between signal and noise (Weidemann and Kahana, 2016).

#### EEG data analysis

Statistical differences in oscillatory activity were investigated using nonparametric permutation tests with cluster correction (Maris and Oostenveld, 2007; Maris, 2012) as implemented in the FieldTrip toolbox (Oostenveld et al., 2011). The nonparametric statistical framework is of particular interest in EEG research since it creates its own sampling distribution, which makes it independent of assumptions about any underlying sampling distribution. The cluster correction was applied to correct for multiple comparisons in the three dimensions electrode site, time bin, and frequency bin. Neighboring electrodes were calculated with the built-in FieldTrip template method, which computed on average 6.9 neighbors per channel. When comparing two conditions in the nonparametric framework (e.g., subsequently remembered trials and subsequently forgotten trials), the membership of data in one condition were randomly shuffled in a first step, and on each data point *t* tests were applied. This procedure was repeated for 1000 times to create a distribution of *t* values for each data point. In a second step, the empirical *t* value was compared with the created distribution at each data point. Data points, which were significantly different from the created distribution and were adjacent in time, space, or frequency dimension were summarized to a cluster and the sum of *t* values within each cluster was extracted. With random permutations, this step was repeated 1000 times to create a distribution of the cluster-based test statistic. Finally, the empirical test statistic was compared with this newly created distribution, and *p* values were calculated, which indicated the percentage of random permutations that resulted in a greater test statistic than the empirical one. Since we did not make assumptions about underlying differences, we applied two-sided *t* tests to our comparisons and adjusted our analyses accordingly. Statistical analyses focused on all EEG channels and in a first step on all frequencies. For further analyses, and in accordance with the postulated hypotheses, we averaged across the frequency bands of interest (theta, 3–7.5 Hz; alpha, 8–13 Hz). A time window of interest for the nonparametric analyses was chosen between 0 and 1.5 s following cue onset.

The nonparametric procedure can be generalized to the comparison of more than two groups, which is equivalent to a nonparametric one-way ANOVA. We used the described framework to investigate the three main effects cue (three levels: TBR, TBF, Neutral), memory outcome (two levels: subsequently remembered or forgotten), and ISI duration (two levels: short and long). In addition to the main effects, the interaction between cue and ISI length was also investigated. To analyze this interaction, we calculated contrasting variables and analyzed these variables with the one-way ANOVA procedure. To analyze the interaction between cue and ISI lengths, we compared TBR_LS_ (TBR_Long_ – TBR_Short_) with TBF_LS_ (TBF_Long_ – TBF_Short_) and Neutral_LS_ (Neutral_Long_ – Neutral_Short_). This 3 × 1 repeated-measures ANOVA is statistically equivalent to running a 3 × 2 repeated-measures ANOVA (Blume et al., 2018). To further explore the interaction effect, data in significant clusters were averaged across time window, frequency band, and statistically significant electrodes of significant clusters, and were investigated with a parametric 3 × 2 repeated-measures ANOVA to analyze specific differences and to statistically control for multiple comparisons. This statistical framework was used for all of the following EEG analyses.

Statistical alpha thresholds for all behavioral and EEG analyses were set at 0.05, and two-sided *t* tests were applied where applicable.

## Results

### Manipulation check

Following debriefing, participants were asked whether or not they followed the memory cues. All participants affirmed this question, meaning that the experimental manipulation worked. In addition, participants were also asked for their strategies to remember and to forget the stimuli. All participants used strategies to increase the likelihood to remember TBR stimuli. Most strategies were to mentally create stories (47.6%) or pictures (19%), to categorize the stimuli (11.9%), or simply to mentally repeat the to-be-remembered words. In contrast, only 21.5% reported having used any strategy to increase the forgetting of TBF words. The only named strategy was to repeat TBR words while TBF words should have been processed. Last, we asked participants about their handling of the Neutral words. In total, 23 participants (54.8%) tried to remember Neutral stimuli using the same strategies as for TBR stimuli and 19 (45.2%) tried to forget them.

### Behavioral results

**Figure 2.**
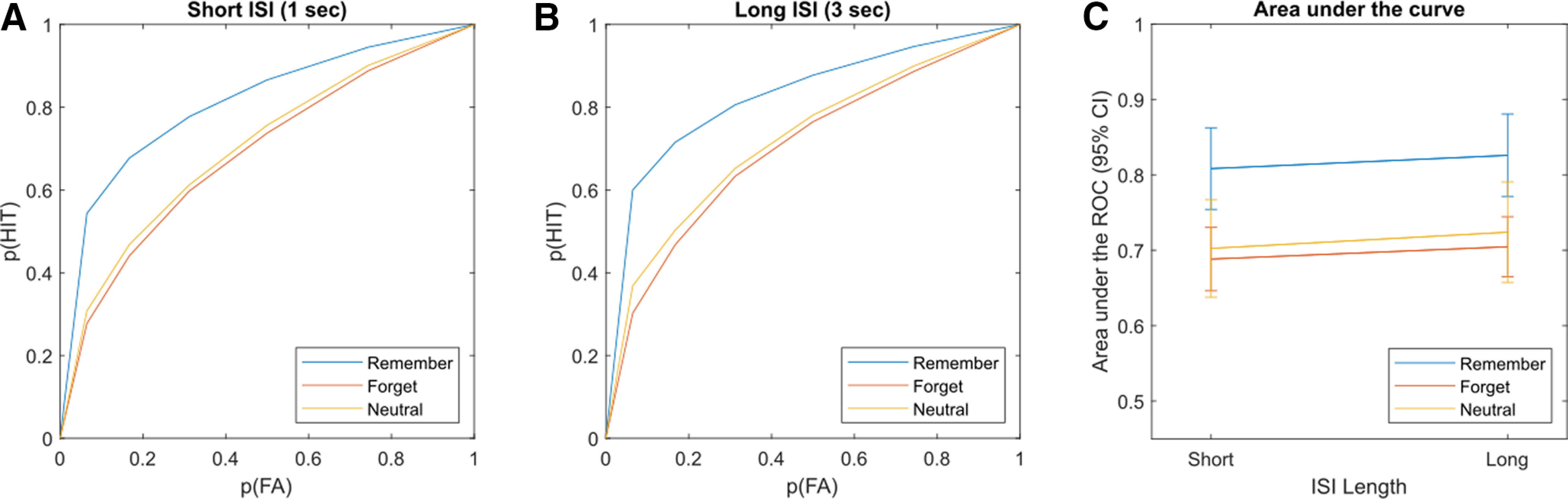
Behavioral results of the experiment. ***A***, Average ROCs for the short ISI (duration = 1 s). Depicted is the relationship between the cumulative increase of hit rate [p(HIT)] and false alarm rate [p(FA)]. The blue line signals the ROC for TBR stimuli, the red line depicts the ROC for TBF stimuli, and the yellow line depicts the ROC for Neutral stimuli. ***B***, Average ROC for the long ISI (duration = 3 s), color coding, and axes are equivalent to those in ***A***. ***C***, Area under the ROC for the three cue conditions and the two ISI durations. Color coding is equivalent to that in ***A*** and ***B***. Error bars indicate the 95% confidence interval for the average area under the ROC.

To investigate differences in recognition performance, a 3 (cues: to-be-remembered, to-be-forgotten, Neutral) × 2 (ISI duration: short, long) repeated-measures ANOVA was conducted. The analysis revealed a significant main effect on the area under the ROC among TBR (mean* *=* *0.82, SD* *=* *0.01), TBF (mean* *=* *0.70, SD* *=* *0.01), and Neutral stimuli (mean* *=* *0.71, SD* *=* *0.02; *F*_(2,82)_ = 77.21, *p *<* *0.001, *η_p_^2^ =* 0.653; Fig. 2*C*). Bonferroni-corrected *post hoc* tests revealed significant differences in the area under the ROC between TBR and TBF (*p *<* *0.001) and between TBR and Neutral stimuli (*p *<* *0.001). TBF and Neutral stimuli were not significantly different (*p *=* *0.324). In addition, the repeated-measures ANOVA revealed a significantly larger area under the ROC for the long ISI (mean* *=* *0.75, SD* *=* *0.01) compared with the short ISI (mean* *=* *0.73, SD* *=* *0.01; *F*_(1,41)_ = 11.73, *p *=* *0.001, *η_p_^2^* = 0.222). In contrast to the two main effects, the interaction between cue and ISI duration was not significant (*F*_(2,82)_ = 0.08, *p *=* *0.921, *η_p_^2^ =* 0.002).

In addition to these analyses, we also examined whether there was a statistical relationship between intentional remembering and intentional forgetting. A correlation analysis revealed that participants with better memory for TBR items showed worse memory for TBF items (short ISI: *r*_(42)_ = −0.509, *p *<* *0.001; long ISI: *r*_(42)_ = −0.599, *p *<* *0.001).

### EEG results

#### Main effect cue

**Figure 3.**
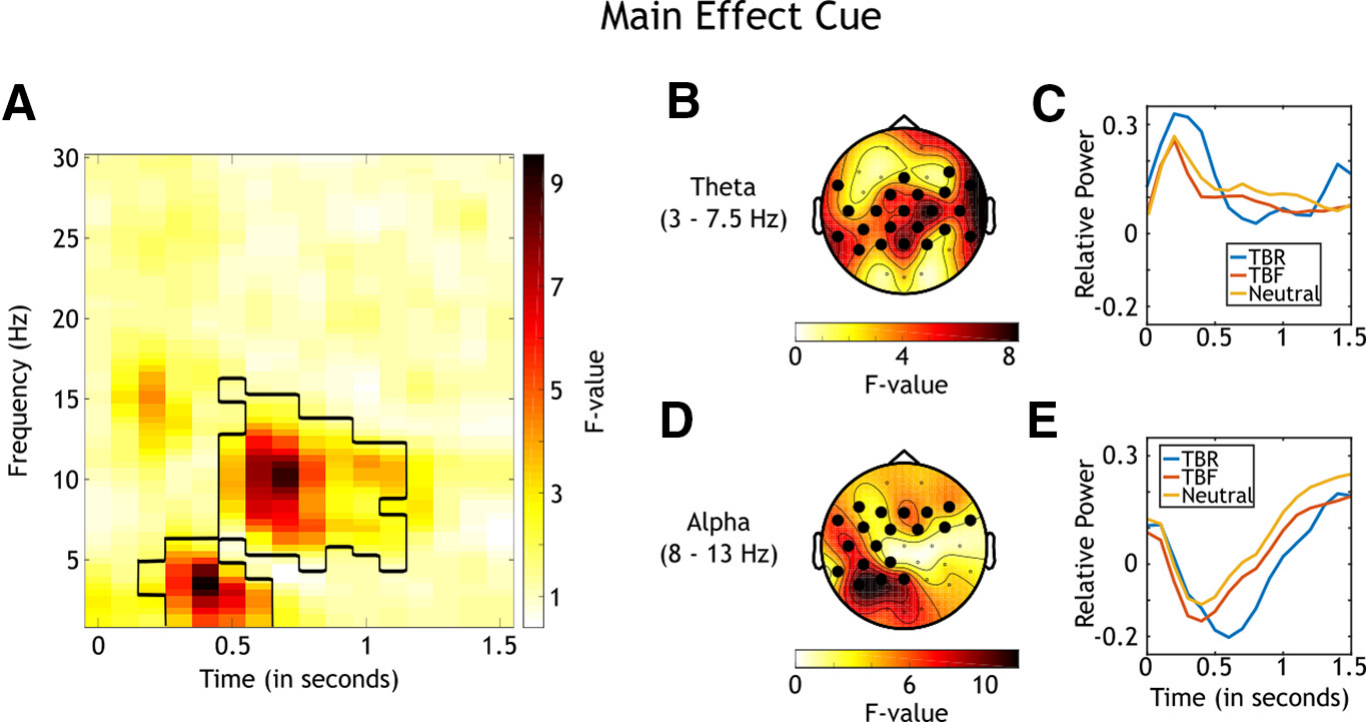
Analyses of the main effect Cue (*N *=* *42). ***A***, *F* values for the nonparametric comparison between time–frequency decompositions of the three cues (TBR, TBF, Neutral). Significant clusters are black rimmed. The analysis was conducted for all electrodes, time points, frequencies, and participants, and the average of all electrodes is plotted. ***B***, Topographical distribution of *F* values of the revealed cluster in theta frequencies averaged across theta band (3–7.5 Hz) and time window of the cluster (0.2–0.5 s following cue onset). Significant electrodes are highlighted. ***C***, Theta power relative to baseline over time averaged across theta frequencies, significant electrodes of the theta cluster, and participants. The blue line signals TBR, the red line signals TBF, and the orange line signals Neutral cue processing. ***D***, Topographical distribution of *F* values of the revealed cluster in alpha frequencies averaged across the alpha band (8–13 Hz) and the time window of the cluster (0.3–1.4 s following cue onset). Significant electrodes are highlighted. ***E***, Alpha power relative to baseline over the time averaged across alpha frequencies, significant electrodes of the alpha cluster, and participants. Color coding is equivalent to that in ***C***.

In accordance with the research hypotheses, we first investigated differences in oscillatory power among the processing of TBR, TBF, and Neutral cues. To account for the differences in trial numbers in these conditions, EEG data were averaged across subsequently remembered and subsequently forgotten trials for each condition. Statistical differences between these averages were then investigated using a nonparametric analysis on the time–frequency decompositions at all frequencies, electrodes, and time points (Fig. 3*A*). The analysis revealed significant clusters in theta frequencies (*p*_corrected_ = 0.003; 0.2–0.5 s following cue onset) and alpha frequencies (*p*_corrected_ < 0.001; 0.5–1.2 s following cue onset). In a second step, nonparametric analyses were conducted based on averages across either theta frequencies (3–7.5 Hz) or alpha frequencies (8–13 Hz). At theta frequencies, the analysis showed a dense central topography averaged across the significant cluster (Fig. 3*B*). For further statistical analyses of this cluster, power for each participant was averaged across theta frequencies, the time window of the cluster, and the significant electrodes of the cluster. Subsequent Bonferroni-corrected *post hoc* tests using this averaged data revealed stronger theta power in processing TBR cues than TBF cues (0.28; 95% CI = 0.17, 0.39; *p *<* *0.001) and also stronger theta power for processing TBR than Neutral cues (0.26; 95% CI = [0.15, 0.38]; *p *<* *0.001). A difference in theta power between TBF and Neutral cues was not evident (−0.02; 95% CI = [−0.10, 0.06]; *p *=* *1). This pattern of results indicated that processing TBR cues recruited the most theta power in relation to processing the other cues.

The cluster in alpha frequencies was characterized by a broad central topography (Fig. 3*D*), and further *post hoc* tests for differences in alpha power between the conditions (averaged across alpha frequencies, time window, and significant electrodes of the cluster) revealed stronger alpha power for TBF cues than for TBR cues (0.09; 95% CI = [0.02, 0.15]; *p *=* *0.004), and for Neutral cues than for TBR cues (0.11; 95% CI = [0.04, 0.17]; *p *=* *0.001). There was no difference in alpha power between the processing of TBF and of that of Neutral items (−0.02; 95% CI = [−0.09, 0.05]; *p *=* *1). This pattern of results indicated that TBR processing recruited the least alpha power in relation to the processing of the other cues.

The reported differences in power between the conditions were also revealed by plotting baseline corrected power values over the time averaged across either theta frequencies (Fig. 3*C*) or alpha frequencies (Fig. 3*E*), participants, and the significant electrodes of the respective cluster. Descriptively, theta power was strongest and alpha power was weakest for TBR processing compared with TBF or Neutral processing.

#### Main effect memory outcome

**Figure 4.**
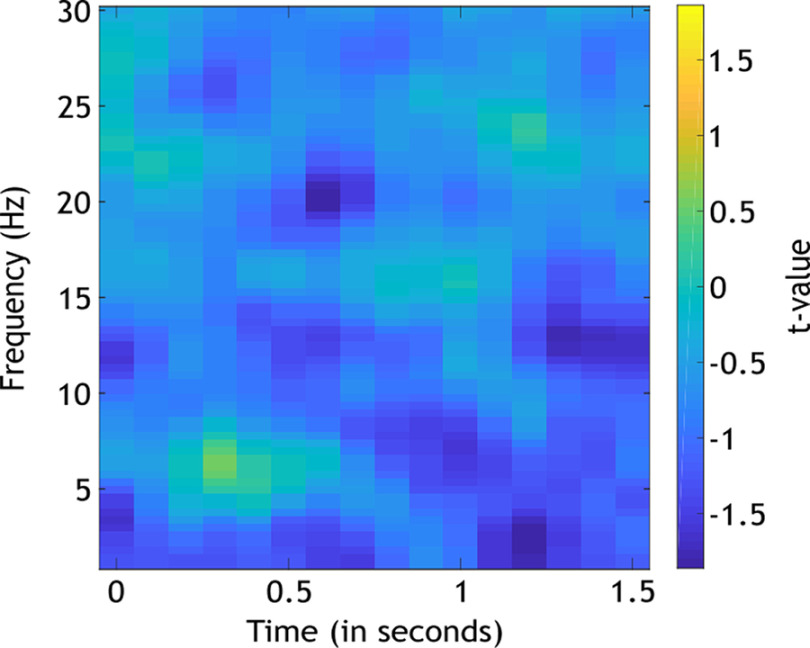
Analyses of the main effect Memory Outcome (*N *=* *42). Plotted are *t* values for the nonparametric comparison between the time–frequency decompositions of the two memory outcomes (subsequently remembered and subsequently forgotten). No significant clusters emerged. The analysis was conducted for all electrodes, time points, frequencies, and participants, and the average of all electrodes is plotted.

The main effect, Memory Outcome, was statistically investigated using a separate, but equivalent averaging procedure. To account for differences in trial numbers, EEG data for subsequently remembered and subsequently forgotten trials were averaged across TBR, TBF, and Neutral cues. Then, a nonparametric analysis at all frequencies, time points, and electrodes was conducted for the comparison between time–frequency decompositions of the subsequently remembered and subsequently forgotten stimuli independent from cue condition (Fig. 4). This analysis revealed significant clusters in neither theta frequencies nor alpha frequencies (lowest *p*_corrected_ = 0.027). Given the absence of any statistical difference between subsequently remembered and forgotten trials, no further processing steps were completed.

#### Difference between subsequently remembered TBR and subsequently remembered TBF stimuli

To investigate whether or not oscillatory activity at theta and alpha frequencies was specifically different for subsequently remembered TBR and subsequently remembered TBF items, we analyzed the contrast between these two conditions. Both of these conditions represented successful encoding, for TBR intentional and for TBF unintentional encoding.

Since nine participants remembered almost all TBR information (see Materials and Methods), these participants were excluded from this analysis, and the number of participants was reduced to *N *=* *33.

**Figure 5.**
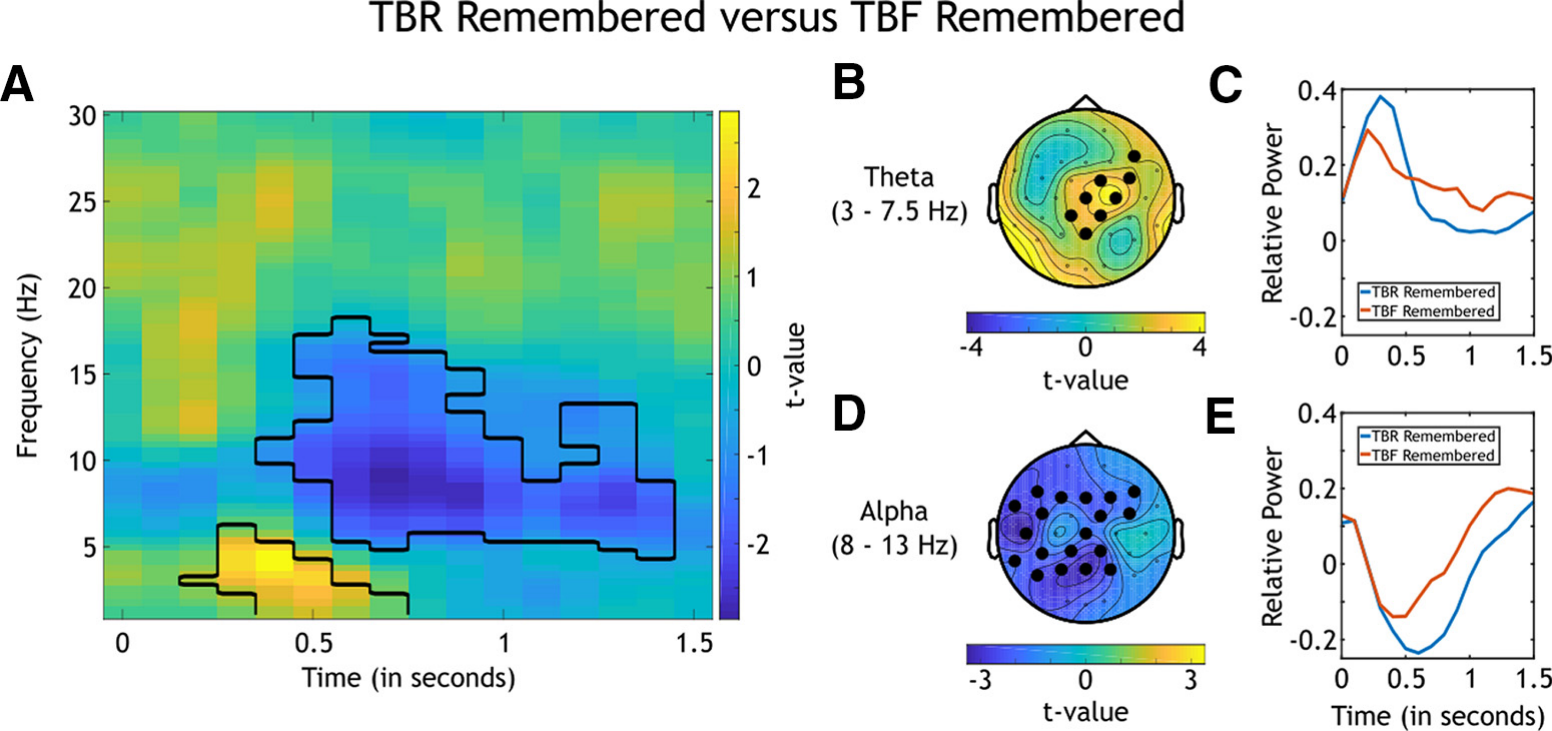
Analyses of the oscillatory differences between subsequently remembered TBR and subsequently remembered TBF stimuli (*N *=* *33). ***A***, *t* values for the nonparametric comparison between time–frequency decompositions of the two combinations. Significant clusters are black rimmed. The analysis was conducted for all electrodes, time points, frequencies, and participants, and the average of all electrodes is plotted. ***B***, Topographical distribution of *t* values of the revealed cluster in theta frequencies averaged across the theta band (3–7.5 Hz) and the time window of the cluster (0.3–0.4 s following cue onset). Significant electrodes are highlighted. ***C***, Theta power relative to baseline over the time averaged across theta frequencies, significant electrodes of the theta cluster, and participants. The blue line signals subsequently remembered TBR, and the red line signals subsequently remembered TBF stimuli. ***D***, Topographical distribution of *t* values of the revealed cluster in alpha frequencies averaged across the alpha band (8–13 Hz) and the time window of the cluster (0.5–1.3 s following cue onset). Significant electrodes are highlighted. ***E***, Alpha power relative to baseline over time averaged across alpha frequencies, significant electrodes of the alpha cluster, and participants. Color coding is equivalent to that in ***C***.

The first step in the comparison of differences in time–frequency decompositions between remembered TBR and remembered TBF information was to run a nonparametric analysis at all frequencies, time points, and electrodes (Fig. 5*A*). This analysis revealed significant clusters in theta frequencies (*p*_corrected_ = 0.019; 0.3–0.4 s following cue onset) and alpha frequencies (*p*_corrected_ = 0.002; 0.5–1.3 s following cue onset).

Running a separate analysis averaged across theta frequencies (3–7.5 Hz) showed a central topography of the theta cluster (Fig. 5*B*). A visual depiction of power over time relative to baseline averaged across theta frequencies, electrodes of the significant cluster, and participants showed that theta power was stronger for subsequently remembered TBR items compared with subsequently remembered TBF items (Fig. 5*C*). A second analysis averaged across alpha frequencies (8–13 Hz) showed a broad central topography of the cluster in these frequencies (Fig. 5*D*). Averaging power relative to baseline across alpha frequencies, electrodes of the cluster, and participants revealed that power in alpha frequencies was weaker for subsequently remembered TBR stimuli compared with subsequently remembered TBF stimuli (Fig. 5*E*).

#### Difference between subsequently forgotten TBF and subsequently forgotten TBR stimuli

Analogous to the previous analysis, we compared subsequently forgotten TBF and subsequently forgotten TBR stimuli to investigate differences between intentional forgetting (in TBF) and unintentional forgetting (in TBR). Participants with <10 trials in one of these conditions were again excluded from the analysis, reducing the sample size to *N *=* *33.

**Figure 6.**
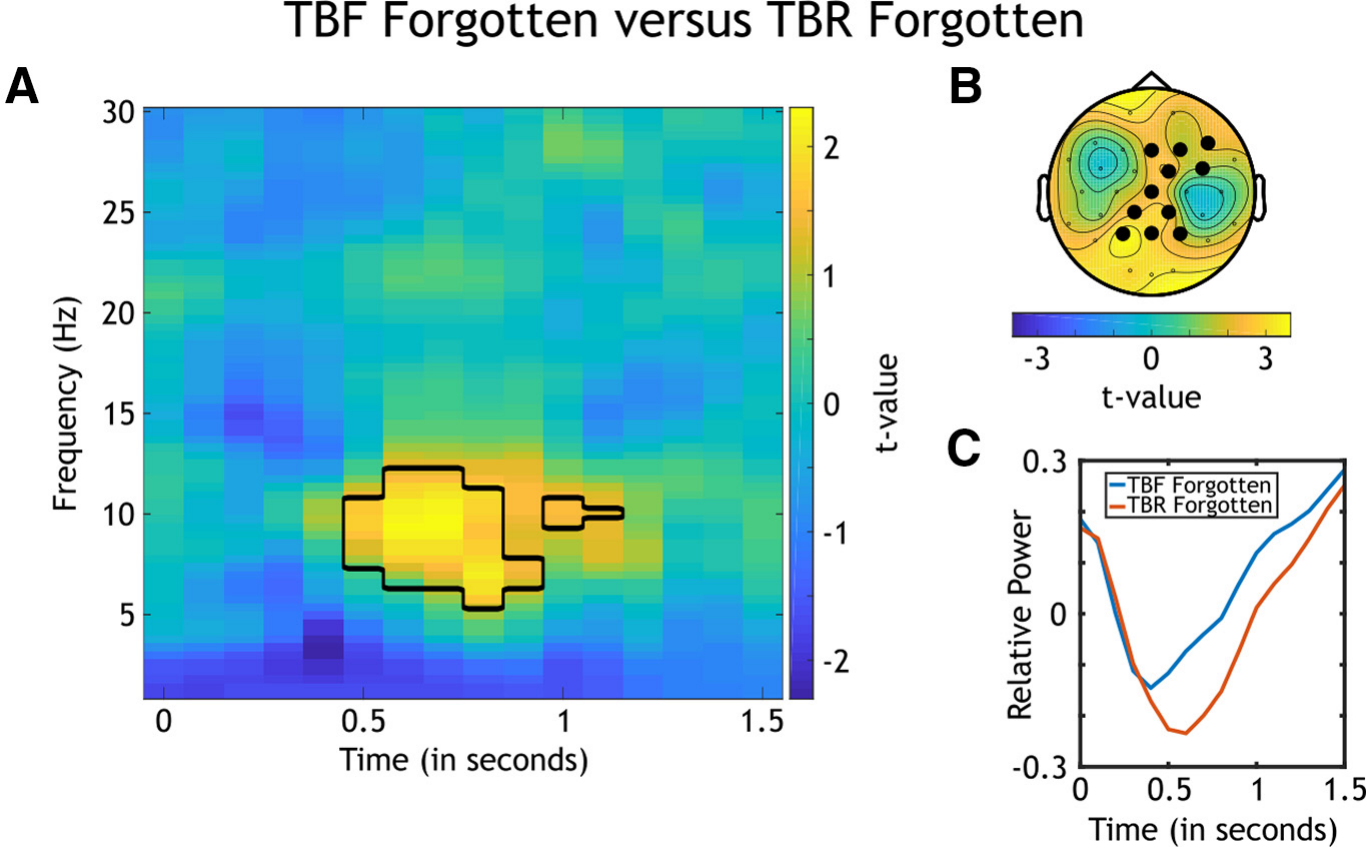
Analyses of the oscillatory differences between subsequently forgotten TBF and subsequently forgotten TBR stimuli (*N *=* *33). ***A***, *t* Values for the nonparametric comparison between time–frequency decompositions of the two combinations. Significant clusters are black rimmed. The analysis was conducted for all electrodes, time points, frequencies, and participants, and the average of all electrodes is plotted. ***B***, Topographical distribution of *t* values of the revealed cluster in alpha frequencies averaged across the alpha band (8–13 Hz) and the time window of the cluster (0.5–0.9 s following cue onset). Significant electrodes are highlighted. ***C***, Alpha power relative to baseline over the time averaged across alpha frequencies, significant electrodes of the theta cluster, and participants. The blue line signals subsequently forgotten TBF stimuli, and the red line signals subsequently forgotten TBR stimuli.

The first step in the comparison of differences in time–frequency decompositions between the two conditions was to run a nonparametric analysis at all frequencies, time points, and electrodes (Fig. 6*A*). This analysis revealed a significant cluster in alpha frequencies (*p*_corrected_ < 0.001; 0.5–0.9 s following cue onset).

Running a separate nonparametric analysis averaged across alpha frequencies (8–13 Hz) showed the dense central topography of the cluster in these frequencies (Fig. 6*B*).

Averaging power relative to baseline across alpha frequencies, electrodes of the cluster, and participants revealed stronger alpha power for subsequently forgotten TBF stimuli compared with subsequently forgotten TBR stimuli (Fig. 6*C*). This result indicated that intentional forgetting (in TBF) recruited more alpha power than unintentional forgetting (in TBR).

#### Interaction between cue and ISI duration

**Figure 7.**
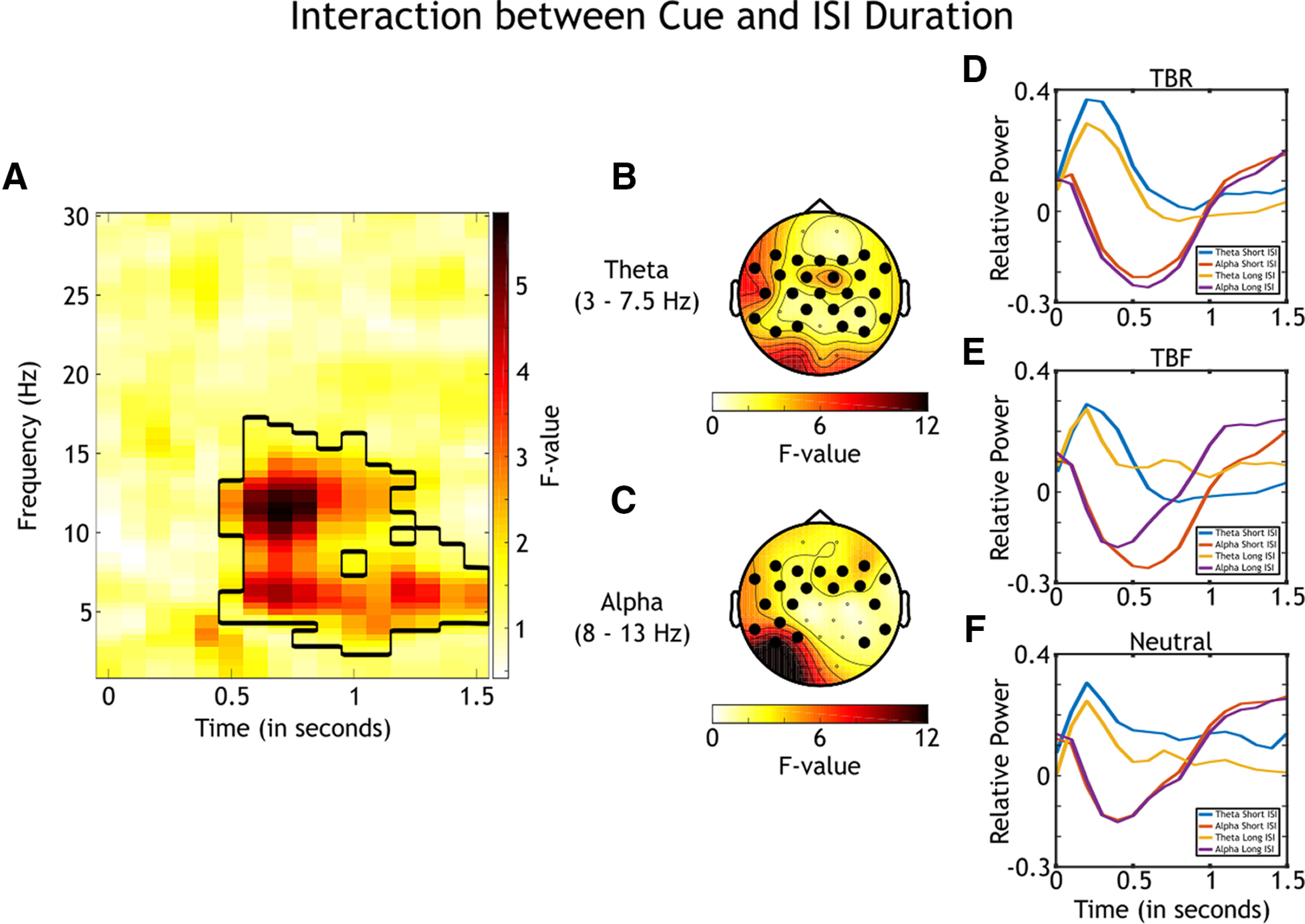
Results for the nonparametric analysis of the interaction between cue and ISI duration (*N *=* *42; variables: TBR_LS_, TBF_LS_, and Neutral_LS_). ***A***, *F* values for the nonparametric comparison between time–frequency decompositions of the interaction variables. Significant clusters are black rimmed. The analysis was conducted for all electrodes, time points, frequencies, and participants, and the average of all electrodes is plotted. ***B***, Topographical distribution of *F* values of the revealed cluster in theta frequencies averaged across the theta band (3–7.5 Hz) and the time window of the cluster (0.7–1.5 s following cue onset). Significant electrodes are highlighted. ***C***, Topographical distribution of *F* values of the revealed cluster in alpha frequencies averaged across the alpha band (8–13 Hz) and the time window of the cluster (0.5–1.3 s following cue onset). Significant electrodes are highlighted. ***D***, Theta and alpha power relative to baseline over time for the processing of TBR cues. Data are averaged across time windows of the clusters, significant electrodes of the clusters, participants, and either theta or alpha frequencies. The blue line signals theta power at the short ISI, the yellow line signals theta power at the long ISI, the red line signals alpha power at the short ISI, and the purple line signals alpha power at the long ISI. ***E***, Theta and alpha power relative to baseline over time for the processing of TBF cues. Color coding and axes are equivalent to those in ***D***. ***F***, Theta and alpha power relative to baseline over time for the processing of Neutral cues. Color coding and axes are equivalent to those in ***D*** and ***E***.

To investigate whether the available processing time mediates the degree of rehearsal and inhibition in the control of memory encoding, we analyzed the interaction of time–frequency decompositions between the presented cue and ISI duration. First, a nonparametric analysis across all frequencies, time points, and electrodes was conducted (Fig. 7*A*). This analysis revealed significant clusters in theta frequencies (*p*_corrected_ = 0.005; 0.7–1.5 s following cue onset) and alpha frequencies (*p*_corrected_ < 0.001; 0.5–1.3 s following cue onset).

Second, separated analyses were conducted averaged across either theta frequencies (3–7.5 Hz) or alpha frequencies (8–13 Hz). For the cluster in theta, the analysis showed a broad central topography (Fig. 7*B*). For the cluster in alpha frequencies, the topography was broad but excluded the central electrodes (Fig. 7*C*).

Power averaged across significant electrodes and participants revealed differences over time between processing the cues and the ISI durations (Figs. 7*D–F*). Descriptively, for TBR, theta power was stronger at the short ISI compared with the long ISI. For TBF, the difference between short and long ISIs seemed to be largest for alpha power. Last, processing of the short and long ISIs was similar for processing of the no-cued items. To statistically analyze these differences, Bonferroni-corrected *post hoc* tests at each cue with data averaged across either theta or alpha frequencies, time window of the significant clusters, and significant electrodes were conducted. For theta frequencies, the analysis revealed that TBR processing recruited stronger power at the short ISI compared with the long ISI (0.1; 95% CI = [0.01 0.19]; *p *=* *0.029). There was no difference in theta power between short and long ISIs for TBF or Neutral processing. The analysis for alpha frequencies revealed stronger power for TBF processing at the long ISI compared with the short ISI (−0.12; 95% CI = [−0.21, −0.03]; *p *=* *0.009) and stronger power for Neutral processing at the short ISI compared with the long ISI (0.09; 95% CI = [0.03 0.16]; *p *=* *0.005). There was no difference in alpha power for TBR processing between the two ISIs.

## Discussion

The aim of the current study was to explore associations between power at theta (3–7.5 Hz) and alpha (8–13 Hz) frequencies and item-method DF, and to test whether the time available to process a memory cue mediates this association. For that purpose, we measured EEG activity while participants performed a DF task in which the time to process TBR and TBF stimuli was varied blockwise.

Consistent with previous research (Basden et al., 1993; MacLeod, 1998; Scholz and Dutke, 2019; Fellner et al., 2020), we found strong behavioral evidence for DF: TBR stimuli were recognized with higher accuracy than TBF or Neutral stimuli. In contrast, no difference in accuracy was found between TBF and Neutral stimuli. Recognition was generally more accurate for items that were followed by a 3 s ISI compared with a 1 s ISI during encoding. Since this advantage in recognition performance was independent of cue condition, participants were able to use additional time to increase recognition for TBR stimuli but were unable to use additional time to increase the forgetting of TBF stimuli. In contrast, the longer ISI duration was associated with even better recognition for TBF stimuli. These results are consistent with research regarding processing time in DF (Wetzel and Hunt, 1977) and suggest that participants might have been aware of strategies to remember TBR stimuli, but were unaware of strategies to increase the likelihood of forgetting TBF stimuli. Indeed, most participants reported not having used any strategies to process TBF stimuli; strategies were only applied to process TBR stimuli. However, we found a significant negative correlation between recognition for TBF and TBR items, indicating beneficial effects of intentional forgetting on remembering. This correlation might be driven by the difference in the application of strategies.

Alpha oscillations (8–13 Hz) have been associated with selective suppression of task-irrelevant information (Foxe and Snyder, 2011; Samaha et al., 2020). Accordingly, studies in DF have revealed stronger alpha power in an earlier time window in response to TBF compared with TBR stimuli, which was interpreted as successful inhibition of TBF items (Fellner et al., 2020). Consistent with these reports, alpha power in the present study was stronger for TBF than TBR processing (0.3–1.4 s following cue onset). In addition, alpha power was also stronger for subsequently forgotten TBF information than for subsequently forgotten TBR information (0.5–0.9 s following cue onset), indicating specific activity in alpha frequencies related to intentional forgetting in DF. This result is direct evidence for the inhibition of to-be-forgotten information.

In contrast to alpha oscillations, theta oscillations (3–7.5 Hz) have not yet been investigated in DF. Hypothetically, they are associated with two different processes in this task: either with the selective rehearsal of information (Klimesch et al., 1996; Lega et al., 2012) or with conflict monitoring and response inhibition (Hanslmayr et al., 2008; Nigbur et al., 2011, 2012; Cohen and Donner, 2013). Our finding of stronger theta power in processing TBR in contrast to TBF items is consistent with the former alternative: TBR cues induced participants to rehearse the last presented stimulus, and this rehearsal process recruited stronger theta power. Theta power was also stronger for subsequently remembered TBR stimuli compared with subsequently remembered TBF stimuli, indicating specific activity for intentional encoding in DF. Related to these effects, previous research revealed stronger theta power during encoding for subsequently remembered items compared with subsequently forgotten items (Düzel et al., 2010; Fellner et al., 2013; Scholz et al., 2017). In contrast to these results, however, we did not find any difference in theta power between subsequently forgotten items and subsequently remembered items independent of the presented cue. Moreover, we did not find any difference in alpha power between subsequently remembered and forgotten information independent from cue condition. This result is in contrast to previous reports that reported stronger alpha power in an earlier time window (0.5–0.9 s following cue onset) for subsequently forgotten information compared with subsequently remembered information (Hanslmayr et al., 2012). The reasons for this absence of effects are currently unclear. However, since prior studies revealed statistical differences using more simple encoding tasks one might speculate that the reported effects do not generalize to more complex encoding tasks like in DF.

Processes of DF might also depend on task characteristics like time available to process a memory cue. Previous behavioral research in DF reported that forgetting was only more resource demanding than remembering when a short processing time was given. For longer processing times, the difference in resource demands vanished (Fawcett and Taylor, 2008). Therefore, the time to process a memory cue might mediate neural processes underlying intentional forgetting. We directly tested this idea and found stronger theta power at central electrodes for TBR at the short ISI (1 s) compared with the long ISI (3 s) and stronger alpha power at central electrodes for TBF at the long compared with the short ISI. Thus, cognitive processes related to these frequency bands were initiated when participants were aware that they would have a longer time to process the memory cue. Shorter processing times, however, did not recruit these oscillatory effects.

In sum, the pattern of results, especially regarding theta and alpha oscillations, indicates that DF can be best explained by a two-process account: faster rehearsal, indicated by increased theta power for TBR at the shorter ISI, and slower inhibition, indicated by stronger alpha power for TBF processing at the longer ISI. Interestingly, the processes associated with theta frequencies were more pronounced when only a short processing time was given. Presumably, when more processing time is available, participants do not engage in these fast cognitive processes that are associated with theta power, but use the longer interval to spread out similar processes over the longer time period. This pattern of results could explain differences between cue processing in the two ISI durations and suggests an influence of processing time on DF.

We did not find any difference in recognition performance or oscillatory activity between TBF and the Neutral condition, perhaps because of the fact that half of the participants reported to have treated Neutral stimuli as TBF stimuli. However, the other half of the sample reported to have treated Neutral stimuli as TBR stimuli, and we found differences between TBR and the Neutral condition in recognition performance and oscillatory activity. Perhaps participants were less motivated to rehearse Neutral items than TBR items, which could explain the pattern of results. Thus, the Neutral condition provided insight into how participants behaved when no precise memory cue was given, which represents important orientation in analyzing processes of remembering and forgetting in DF. As an alternative strategy, future research could potentially include other control conditions that restrict the participants’ behavior in theoretically meaningful ways to disentangle processes of intentional and unintentional remembering and forgetting.

Although the present results demonstrate a strong correlation between oscillatory activity in theta and alpha bands and DF performance, directly influencing power in these bands during DF could be the next step to test for a potential causal relationship. Neurofeedback or noninvasive brain stimulation could be applied to modulate power in frontal (Enriquez-Geppert et al., 2013) or central (Vernon et al., 2003) theta frequencies following stimulus presentation. Based on the present results, this procedure should increase remembering, specifically of TBR information. Similarly, modulating power in alpha frequencies following stimulus presentation should increase forgetting of TBF items (Hanslmayr et al., 2005; Zoefel et al., 2011). Furthermore, neural sources of the oscillatory effects here reported need to be investigated with spatially more sensitive methods, like the simultaneous recording of EEG and fMRI data (Huster et al., 2012; Lewis et al., 2016). Establishing these causal relationships and revealing the neural sources for the reported effects could further improve the understanding of neural processes underlying memory control in DF.

To conclude, this study revealed a strong association between oscillatory activity in the theta and alpha frequency bands and performance in item-method DF, which is consistent with a processing time-dependent account: processes associated with theta and alpha power were enhanced when only a short processing time was given, implying that the strategies used in DF depend on the time available for processing memory cues.

## Synthesis

Reviewing Editor: Satu Palva, University of Helsinki Decisions are customarily a result of the Reviewing Editor and the peer reviewers coming together and discussing their recommendations until a consensus is reached. When revisions are invited, a fact-based synthesis statement explaining their decision and outlining what is needed to prepare a revision will be listed below. The following reviewer(s) agreed to reveal their identity: Paul Sauseng, Marie-Christin Fellner.

In this manuscript the authors investigate the oscillatory power correlates of item method directed forgetting. This is a nice piece of research. Particularly, the clarity of the introduction and how it motivates the hypotheses is excellent. The hypotheses themselves are well described and make perfect sense.

The manuscript is generally well written, but however results and statistical analysis are lacking in depth description and some result figures seem erroneously labeled.

Major points:

1. Statistical analysis: Most serious concern is how the results are described and plotted. There are some serious errors and unclearly described analyses that render it hard to jugde the quality of the reported results. The outlined procedure of splitting a 3x2 Anova in F and t test is to the best of my knowledge valid and well described in the methods. The presentation of the results however is lacking precision and figure 3 and 6 are wrongly labelled as t values, while the reported test here are F-tests. If this is not the case, proper F-tests should be run for the interactions and the 3-level factor main effects.

2. Authors mention that they plot and base the post hoc test on t"he most prominent electrodes”. This sounds fishy/doubledipped. The selection of electrodes needs to be based on an independent criteria, i.e. all electrodes of the significant F-test cluster for post hoc tests between the three conditions.

3. The TF plots in all the figures are not well described. What is shown here? Average of all electrodes? The marked significant clusters and what is shown in the figure does not match (for example 5A). It is not outlined how the cluster test was run, was clustering employed across spatial and temporal dimensions (averaging across frequencies)? The „real” results i.e. the significant cluster should then be plotted. Consider plotting the contour pf the significant cluster on the topoplots and TF plots.

4. As f-value plots show no direction of effects additional plot should depict timecourses of alpha/theta power of each condition and/or topoplots should show absolute values.

5. Colorbars should be symmetrical for t-values to enable interpretation of the topographies

6. Another concern are the trial numbers/exclusion criteria. The authors report no measures of preprocessing except ICA (i.e. no visual check of data or excluding trials with artifacts). Considering the low trial numbers in each condition (240/ 3 cue conditions /2 memory conditions / 2 isi cond), a few noisy trials can seriously influence the results. Considering the trial number, how many trials were in each condition? Based on which criteria was a trial scored remembered or forgotten?

7. Memory inhibition effects are very sensitive to instructions. What was the concrete instruction given to the participants prior to the experiment? Related to the problem of instruction is also, the critique concerning the control condition which does not really serve as a control here. The authors nicely show using a post experimental questionnaires, that participants basically did whatever they wanted after a control cue. This behavior renders the „control” condition a in fact „not at all controlled” condition. Therefore, it would be better dropping the control condition from the analysis and simplifying the analysis.

8. Baseline of power effects: The authors use the epoch of -500 to 0 sec prior to the cue as baseline. This is potentially problematic, as this includes the cue onset, especially theta effects around the onset might smear to the baseline window. Furthermore, the pre cue baseline is not a true baseline insofar as it functions as the maintenance interval of the item. It is likely that memory effects are already present pre cue and using this time window as baseline may transfer these effects post cue I have merely one major concern which is the thresholding of the interaction effects.

It is problematic that thresholding of the interaction effects is at p<.1. which is a strange threshold at the 10% level. Thresholds of p<.005 would ascertain that there will be fewer false positives in research (and consequently stronger effects should be easier to reproduce). The current approach leads to effects as shown in Fig 7 - effects that just cannot be seen. Hence, the interaction effects should better be thresholded at p<.05 as well.

Minor suggestions:

a) The terms “processes” and “processing” might have been used a bit too excessively in the abstract.

b) in Fig 2 it would be nice to have the line colours described directly in the figure, not only in the legend.
